# Voltage-Gated Proton Channel Hv1 Regulates Neuroinflammation and Dopaminergic Neurodegeneration in Parkinson’s Disease Models

**DOI:** 10.3390/antiox12030582

**Published:** 2023-02-25

**Authors:** Matthew L. Neal, Eric E. Beier, Muhammad M. Hossain, Alexa Boyle, Jiaying Zheng, Chunki Kim, Isha Mhatre-Winters, Long-Jun Wu, Jason R. Richardson

**Affiliations:** 1 Department of Environmental Health Sciences, Robert Stempel College of Public Health and Social Work, Florida International University, Miami, FL 33199, USA; 2 Environmental and Occupational Health Sciences Institute, Rutgers University, Piscataway, NJ 08854, USA; 3 Department of Pharmaceutical Sciences, Center for Neurodegenerative Disease and Aging, Northeast Ohio Medical University, Rootstown, OH 44201, USA; 4 Department of Neurology, Mayo Clinic, Rochester, MN 55905, USA

**Keywords:** Parkinson’s disease, neuroinflammation, Hv1, HVCN1, microglia, MPTP, LPS

## Abstract

Although the precise mechanisms for neurodegeneration in Parkinson’s disease (PD) are unknown, evidence suggests that neuroinflammation is a critical factor in the pathogenic process. Here, we sought to determine whether the voltage-gated proton channel, Hv1 (HVCN1), which is expressed in microglia and regulates NADPH oxidase, is associated with dopaminergic neurodegeneration. We utilized data mining to evaluate the mRNA expression of *HVCN1* in the brains of PD patients and controls and uncovered increased expression of the gene encoding Hv1, *HVCN1,* in the brains of PD patients compared to controls, specifically in male PD patients. In an acute 1-methyl-4-phenyl-1,2,3,6-tetrahydropyridine (MPTP; 4 × 16 mg/kg) mouse model of PD, *Hvcn1* gene expression was increased 2-fold in the striatum. MPTP administration to wild-type (WT) mice resulted in a ~65% loss of tyrosine hydroxylase positive neurons (TH^+^) in the substantia nigra (SN), while a ~39% loss was observed in Hv1 knockout (KO) mice. Comparable neuroprotective effects of Hv1 deficiency were found in a repeated-dose LPS model. Neuroprotection was associated with decreased pro-inflammatory cytokine levels and pro-oxidant factors in both neurotoxicant animal models. These in vivo results were confirmed in primary microglial cultures, with LPS treatment increasing *Hvcn1* mRNA levels and Hv1 KO microglia failing to exhibit the LPS-mediated inflammatory response. Conditioned media from Hv1 KO microglia treated with LPS resulted in an attenuated loss of cultured dopamine neuron cell viability compared to WT microglia. Taken together, these data suggest that Hv1 is upregulated and mediates microglial pro-inflammatory cytokine production in parkinsonian models and therefore represents a novel target for neuroprotection.

## 1. Introduction

The main pathophysiological hallmark of Parkinson’s disease (PD) is the selective loss of dopaminergic neurons in the substantia nigra (SN) region of the brain [1]. Although there are drugs that can alleviate some symptoms of PD, there are no viable therapeutic options that successfully lead to neuroprotection without dangerous off-target effects. Several pre-clinical and clinical studies have been tested for neuroprotection in PD [2], including a prominent study based on the ability of the tetracycline antibiotic minocycline to reduce neuroinflammation and microglial activation [3]. However, to date, no therapeutic has been developed to reduce neuroinflammation in PD sufficiently.

The precise mechanisms regulating neuroinflammation in PD are not well established. The SN contains a higher proportion of microglia, the resident immune cells of the brain, than other brain regions [4]. Increased numbers of activated microglia are also observed in the post-mortem brains of humans who developed Parkinsonian syndrome after accidentally injecting 1-methyl-4-phenyl-1,2,3,6-tetrahydropyridine (MPTP) decades earlier [5], suggesting that an initial insult to the brain causes a sustained neuroinflammatory environment. Microglial activation, as defined by MAC-1 staining, cytokine induction, and a round, ameboid-like morphology, is consistently observed in the brains of PD patients [6,7,8]. Similar findings are also observed in animal models of PD, including those employing MPTP, lipopolysaccharide (LPS), 6-hydroxydopamine (6-OHDA), and paraquat (PQ) [9,10,11,12]. Thus, specifically targeting neuroinflammation and activated microglia may be a fruitful avenue for neuroprotection in PD.

Unfortunately, targeting neuroinflammation in PD animal models and clinical trials has produced mixed results. The antibiotic minocycline is the most prominently studied drug to reduce neuroinflammation in PD. Early studies reported that minocycline reduced dopaminergic neurodegeneration in the MPTP model through dampening microglial activation, possibly through inhibition of microglial NADPH oxidase [13]. NADPH oxidase is a key player in the generation of reactive oxygen species (ROS) and cytokine release in PD patients and animal models [7,14]. Furthermore, mice lacking NADPH oxidase or its catalytic subunit gp91phox exhibit reduced microglial activation and neurodegeneration following MPTP and LPS administration [13,15]. NADPH oxidase inhibitors also decrease microglial activation and degeneration of dopamine neurons following 6-OHDA and MPTP administration [16,17]. However, minocycline exacerbated MPTP toxicity in both mice and monkeys [18,19]. Likewise, previous clinical trials for minocycline in other neurodegenerative diseases, such as ALS, were stopped because of disease acceleration [20]. A pilot clinical trial of minocycline administration in early-stage PD also identified decreased patients’ tolerability to minocycline [21], but results of a larger clinical trial of efficacy in PD have not been published.

A potential reason that drugs targeting neuroinflammation, particularly microglial activation, may display equivocal results is that their pharmacological targets are not unique to microglia. For example, minocycline may also target non-microglial cells and thus have side effects inducing fever, staining of the teeth, lupus-like disorder, gastrointestinal disturbances, and photosensitivity [22,23,24,25,26]. In addition, isoforms of NADPH oxidase expressed systemically may reduce the effectiveness of inhibitors and cause deleterious off-target or exaggerated pharmacological effects [27]. Thus, identifying cell-specific regulators of NADPH oxidase could represent a significant advance in targeting microglial NADPH oxidase in neurodegeneration.

The hydrogen voltage-gated channel 1 (HVCN1) is a proton-sensitive channel and exchanger that is highly expressed in microglia [28,29,30]. The *Hvcn1* gene encodes a voltage-gated proton channel Hv1 composed of two voltage sensing domains [31]. Microglial Hv1 channels compensate for intracellular acidosis induced by NADPH oxidase during an immune response, thereby sustaining ROS levels that may lead to neuronal damage and neuroinflammation [32,33]. Wu and colleagues previously reported that mice lacking the Hv1 channel were protected from NADPH oxidase-mediated ROS formation and neuronal death in a stroke model [34]. Similarly, Hv1 deficiency reduced demyelination in a model of multiple sclerosis [35]. Further, Hv1 is required for optimal NADPH oxidase activity [36], suggesting that it may be an ideal target for reducing microglial activation and subsequent neurodegeneration. Here, we report that mice lacking Hv1 are protected from the dopaminergic neurotoxicity of MPTP and LPS, which is associated with dampened microglial activation and decreased production of pro-inflammatory cytokines.

## 2. Materials and Methods

### 2.1. HVCN1 Expression Analysis in GEO Datasets

The gene expression omnibus (GEO) is a repository for functional genomics data run by the National Center for Biotechnology Information (NCBI) [37] (https://www.ncbi.nlm.nih.gov/gds (accessed on 1 January 2020)), where investigators can upload high-throughput genomics data. Data were compiled from five different studies that generated gene expression profiles by microarray in the brains of PD patients (*n* = 39 males and 22 females) compared to controls (*n* = 22 males and 14 females) stored as GEO datasets GDS2821 [38], GDS3128 [39,40], GSE49036 [41], GSE43490 [42], and GSE42966.

### 2.2. Animal Care and Treatment Paradigms

Ten to twelve-week-old C57BL/6J male mice from Jackson Laboratories served as the wild type (WT). Global Hv1 KO mice were generated from an in-house breeding colony at Rutgers University or Northeast Ohio Medical University. The generation of Hv1 KO mice has been described previously [34]. Three to four mice were housed per cage in a standard 12 h light/dark cycle at 22 ± 2 °C and 50 ± 10% relative humidity.

Since the risk of developing PD is twice as high in males than in females [43,44], male mice were specifically chosen to study Hv1-mediated effects in animal models of PD. The sample size for each experiment was based on an analysis of statistical power [45]. Briefly, to compute sample size for continuous variables, we obtained an estimate of the population standard deviation of the variable (s) and the magnitude of the difference (d) that we expect to detect, and the sample size is given by *n* = 1 + 2C(s/d)2. C is a constant dependent on α and β and equals 10.51 when α = 0.05 and 1 − β = 0.90.

Mice were randomly assigned to control and treatment groups for each dosing paradigm. For the acute MPTP (Sigma-Aldrich, St. Louis, MO, USA; cat #: M0896-100MG) treatment, mice were injected intraperitoneally (i.p.) every 2 h for a total of 4 doses of either 10 or 16 mg/kg MPTP in 100 μL PBS. Mice were sacrificed 2 or 7 days after the last treatment. For the repeated dose LPS model, 100 μL of LPS (*Salmonella abortus equi S*-form, Enzo Life Sciences, Farmingdale, NY, USA; cat #: ALX-581-009-L002) at a dose of 1 mg/kg in 100 μL PBS was administered i.p. once a day for 4 consecutive days [46]. Animals were sacrificed 1 day or 2 weeks after the last dose of LPS. All studies complied with the ARRIVE guidelines and were carried out in accordance with the NIH Guide for the Care and Use of Laboratory Animals [47]. Experiments were approved by the animal care committees of Rutgers-Robert Wood Johnson Medical School and Northeast Ohio Medical University.

### 2.3. Tissue Preparation

Animals were euthanized with CO_2,_ and brains were removed on ice. The striatum from the left hemisphere was dissected out and frozen in liquid nitrogen. The right hemisphere of the forebrain and the entire hindbrain were drop-fixed in 4% paraformaldehyde at 4 °C for 7 days and then transferred to 30% sucrose for cryoprotection. Coronal sections were cut at 40 μm thickness on a freezing sliding-arm microtome and stored in a cryoprotectant solution containing 25% ethylene glycol and 25% sucrose in PBS at −20 °C [48,49].

### 2.4. Primary Microglial Culture and Treatments

Primary microglia were isolated from whole brains of neonatal (0–3 days old) C57BL/6J wild-type mice or Hv1 KO mice as described previously [50,51,52]. Briefly, brains from mouse pups were collected and gently triturated into a single-cell suspension, then plated in 75 cm^2^ flasks in 10% fetal bovine serum DMEM/F12 supplemented with penicillin (100 U/mL), streptomycin (100 μg/mL), 2 mM _L_-glutamine, 100 μM non-essential amino acids, and 2 mM sodium pyruvate (Invitrogen, Carlsbad, CA, USA). Media was changed 5 days after initial plating, and microglia were separated using a previously published technique using a CD11b magnetic bead isolation kit [52]; (STEMCELL Technologies, Vancouver, BC, Canada; cat #: 18970). This kit labels CD11b-positive cells with magnetic beads, and when put into a magnet, the CD11b-positive cells are retained while other cell types are washed away, creating a >95–97% pure microglial culture. Each isolate was derived from a separate litter obtained from different parents.

Primary microglial cultures were treated with 100 ng/mL of LPS (*Escherichia coli* O111:B4, Sigma, St. Louis, MO, USA; cat # L4391) in DMEM/F12 media with 2% FBS for 6 or 10 h to investigate gene expression changes and protein changes, respectively, and 24 h for conditioned media experiments. The conditioned media experiments were performed in 96-well plates with 150 μL media. Following treatment, the media was collected and briefly centrifuged to remove cellular debris and directly added to the rat N27 dopaminergic neuronal cell line.

### 2.5. Reactive Oxygen Species and Nitric Oxide Quantification

The fluorescent dye CM-H_2_DCFDA was used to determine intracellular reactive oxygen species (iROS). WT or Hv1 KO primary mouse microglia were seeded into black-walled, clear-bottom 96-well plates and incubated with the dye in HBSS for 1 h. Next, the cells were washed thrice with HBSS media to remove any residual dye. The cells were then treated with LPS 100 ng/mL. Fluorescent intensity was measured once every 30 min for 6 h on a SpectraMax M5 plate reader. The fluorescent values for four wells were averaged for each plate. *n* = 3–4 isolations.

The Griess colorimetric assay was chosen for the nitric oxide (NO) determination. WT or Hv1 KO primary mouse microglia were seeded into 96-well plates and treated with no treatment or LPS 100 ng/mL for 24 h. Following the treatment, the media was collected and run using a standard curve from the kit according to the manufacturer’s directions, as described previously [53]. The measured absorbance was compared to the standard curve to give the total concentration of media nitrite. Values were averaged for four wells per plate to produce one biological replicate (*n* = 3–4 isolations).

### 2.6. N27 Cell Culture and Conditioned Media Experiments

N27 (RRID:CVCL_D584) cells were purchased from Millipore Sigma (cat # SCC048), Burlington, MA, USA. N27 cells were grown in RPMI 1640 media supplemented with 10% FBS, 100 U/mL penicillin, 100 µg/mL streptomycin, and 2 mM L-glutamine. For the conditioned media experiments, 5000 N27 cells were seeded into each well of a 96-well plate the night before conditioned media experiments were started. One hundred and fifty microliters of the conditioned media was added to each well for 24 h. The MTS assay was performed by adding 30 μL of the MTS reagent to each well. The assay measures the levels of a colored formazan dye generated by the conversion of the MTS tetrazolium compound by metabolically viable mammalian cells. The plate was read using a SpectraMax M5 plate reader with an absorbance of 490 nm every hour for 4 h. The values were averaged for each treatment group for each plate. *n* = 3 passages.

### 2.7. Immunohistochemistry and Immunocytochemistry

Free-floating brain sections were washed with PBS and steamed in 0.1 M citrate buffer (pH 6.0) for 5 min for antigen retrieval. Sections were incubated in Background Buster (INNOVEX Biosciences, cat #: NB306), followed by overnight incubation with primary antibodies (Hv1 [Rabbit, 1:200, Abcam; cat # ab117520], tyrosine hydroxylase [TH 1:1000, Millipore; cat # ab152], TNF-α [1:750, Abcam, catalog #AB9348], gp91phox [1:500, Santa Cruz; cat # sc-5827], or ionized calcium-binding adapter molecule 1 [Iba1, Wako Diagnostics, 1:850, catalog #019-19741]) in 2% BSA. After rinsing, sections were incubated with an AlexaFluor Plus secondary antibody for Hv1 immunofluorescence (Donkey anti-Rabbit 488, 1:1000; Thermo-Fisher; cat # A32790) or highly cross-adsorbed Alexa Fluor secondary antibodies (Thermo-Fisher, 1:1000–1:2000) for 1 h at room temperature. Sections were rinsed, mounted on slides, and coverslipped with ProLong Gold reagent with or without DAPI (Life Technologies). TH stained sections were imaged on a fluorescent microscope (Zeiss AxioObserver D1, Carl Zeiss) equipped with AxioVision software. Confocal z-stack images were acquired from a Biovision spinning disk microscope comprised of an upright Zeiss AxioImager Z1 microscope equipped with a Yokogawa CSUX1-5000 spinning disc. Images were collected under identical parame ters and reconstructed using Fiji software [54]. At least three sections were analyzed for each animal (*n* = 4–5 mice).

Immunocytochemistry for the primary microglia was performed as previously described [50], and cells were plated onto 8-well chamber slides (Corning; cat. #: 354118) coated with 0.1% poly-D-lysine. After the cells were treated for 10 h, 4% paraformaldehyde was used to fix the cells for 20 min, followed by a PBS wash. Blocking buffer containing 2% BSA, 0.5% Triton X-100, and 0.05% Tween-20 was added to the wells for 1 h. The cells were incubated with primary antibodies iNOS (1:500, Santa Cruz, cat # SC650) or TNF-α (1:750), with Iba1 (1:850) in 2% BSA at 4 °C overnight. An Alexa Fluor fluorescent dye-conjugated secondary antibody in 2% BSA was added and incubated at room temperature on a shaker for 1 h. After washing, coverslips were added to the slides using ProLong GOLD mounting media containing DAPI nuclear stain. Cells were imaged using an Olympus FSX imaging instrument. Integrated fluorescence intensity was measured using the ImageJ software to threshold the fluorescence and select Iba1-positive cells for analysis. A minimum of 50 Iba1-positive cells were analyzed from each isolation (*n* = 3–4 isolations).

### 2.8. Unbiased Stereology

For neuronal quantification, slides were either probed for immunofluorescence with a goat anti-rabbit secondary antibody conjugated to a 594 fluorophore (Invitrogen, cat #: A-11037) or DAB (3,3′-diaminobenzidine) immunostained for TH (1:1000) according to our previous studies [46,49]. The SN was defined based on TH^+^ cells, as outlined previously [49,55,56,57]. TH^+^ cells were only counted if the entire nucleus was within the region or touching the right and top borders. A counting frame of 50 μm × 50 μm with a framing space of 200 μm and a height of 10 μm was chosen. Only the cells that came into focus within the counting frame height were counted. The coefficient error for all animals was equal to or below 0.1, and a minimum of 100 markers were counted within 40–60 framing sites for each animal. Unbiased stereology was performed using a Leica DM2500 (Leica Microsystems, Chicago, IL, USA) and Stereologer computer-assisted stereology software (Stereology Resource Center, St. Petersburg, FL, USA). The SN was delineated using previously described criteria [58] at low magnification. Every sixth section throughout the SN was sampled at higher magnification (63×) with the optical fractionator probe for a total of six sections. For MPTP exposure studies, *n* = 5 for the saline and MPTP groups included both wild-type and Hv1 KO mice. For LPS exposure, *n* = 4 for saline and *n* = 5 for LPS exposure for both wild-type and Hv1 KO mice.

### 2.9. Quantitative Real-Time Polymerase Chain Reaction (qRT-PCR)

RNA was isolated from the striatum (*n* = 4–6 mice per group) using the IBI isolate reagent according to the manufacturer’s directions (IBI Scientific, Peosta, IA, USA, cat #: IB47601). Reverse transcription was performed using the All-in-One cDNA Synthesis SuperMix (Bimake, Houston, TX, USA, cat #: B24408). Genes of interest were normalized to housekeeping gene *Rpl13a* expression. Primer sequences are provided in Supplemental Table S1.

Six hours after treating primary microglial cultures, treatment media was removed and IBI isolate was added to the wells to lyse the cells. RNA was isolated according to the manufacturer’s directions. RT-PCR was performed using the All-in-One cDNA Synthesis SuperMix to convert the RNA into cDNA. Expression levels were determined using RT- PCR with Bimake RT2 SYBR Green Master mix (cat #: B21203) and using previously published primer sets. Gene expression was normalized by the housekeeping genes *Gapdh* and *Rpl13a*. Primer sequences are provided in Supplemental Table S1. The amount of each template was optimized empirically to maximize efficiency without inhibiting the PCR reaction. According to the manufacturer’s guidelines, dissociation and melting curves were run to ensure that single amplicon peaks were obtained without non-specific amplicons. Results are reported as fold-change in gene expression, determined using the delta-delta Ct (ΔΔ^Ct^) method using the threshold cycle (Ct) value for the housekeeping gene and the respective gene of interest in each sample (*n* = 3–4 isolations).

### 2.10. Data Analysis

Data analyses were performed using the Prism 7.04 software package (GraphPad Software, San Diego, CA, USA). Data were assessed for normality by the D’Agostino–Pearson normality test and the Shapiro–Wilk normality test. The data were analyzed using a two-way analysis of variance (ANOVA), followed by Tukey’s multiple comparison test to compare treatment groups in both mouse strains. Differences of *p* < 0.05 were considered statistically significant. The Student’s *t*-test was used when only two groups were being compared, other than the GEO datasets, in which the Mann–Whitney non-parametric test was conducted because the data did not follow a normal distribution. All in vitro experiments were performed with at least three isolations (biological replicates) from at least two independent experiments (technical replicates).

## 3. Results

### 3.1. Hv1 Levels Are Increased in the Brains of PD Patients, Animal Models of PD, and Cultured Microglia following MPTP or LPS Treatment

**Figure 1:**
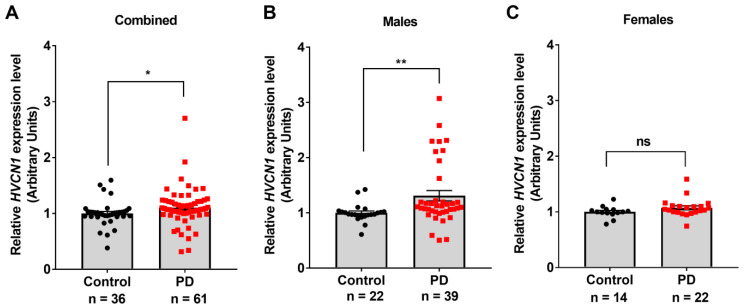
Increased *HVCN1* expression in the brain of PD patients. (**A**) *HVCN1* expression levels are increased in the substantia nigra of PD patients (*n* = 39 males and 22 females) compared to age-matched controls (*n* = 22 males and 14 females). (**B**) *HVCN1* expression levels in male PD patients compared to male controls from the same studies. (**C**) *HVCN1* expression levels in female PD patients compared to female controls from the same studies. GEO numbers were normalized to 1 to generate relative expression values and allow comparison between datasets. Datasets were analyzed by Mann–Whitney non-parametric test. Asterisks denote statistically significant differences—* *p* < 0.05, ** *p* < 0.01, and ns = not statistically significant. Error bars denote the standard error of the mean.

Recent studies in animal models of stroke and multiple sclerosis showed that Hv1 activity and expression increased inflammation and neurodegeneration [34,35]. Therefore, we hypothesized that Hv1 could be involved in the pathogenesis of PD. First, we determined whether expression of the Hv1 gene, HVCN1, was increased in the brains of PD patients compared to age-matched controls. Data were compiled from five studies that generated microarray gene expression profiles in the brains of PD patients (*n* = 39 males and 22 females) compared to controls (*n* = 22 males and 14 females) stored as GEO datasets GDS2821 [38], GDS3128 [39,40], GSE49036 [41], GSE43490 [42], and GSE42966. We examined the gene expression of HVCN1 in these samples and found that PD patients expressed significantly higher mRNA levels of HVCN1 compared to controls, with over 10% increased expression (Figure 1A). Further, separating the data by sex and comparing control males with PD males uncovered a 30% increase in HVCN1 expression level in PD males compared to control males (Figure 1B), whereas there was no difference between PD females and control females (Figure 1C).

**Figure 2:**
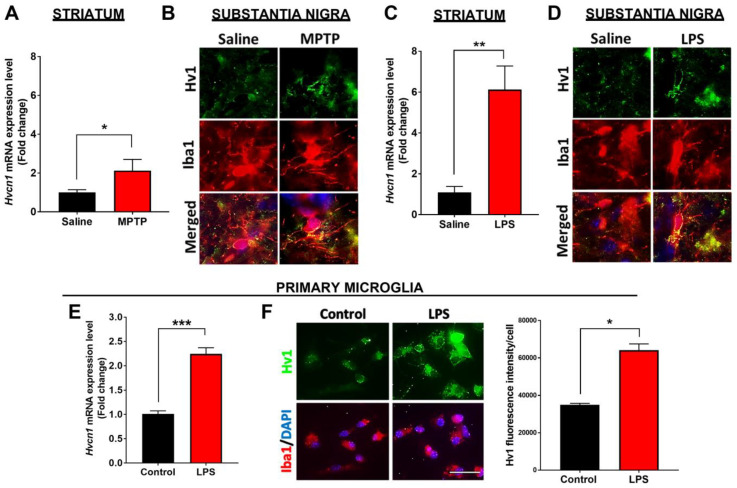
Increased Hv1 levels in the brain of neurotoxic animal models of PD and LPS-treated primary microglia. (**A**) mRNA levels of *Hvcn1* in the striatum of acute MPTP (4 × 10 mg/kg) or saline-treated C57BL/6J mice. (**B**) Representative immunofluorescent images of Hv1 (green) protein level in Iba1-positive (red) cells in SN of saline and MPTP treated C57BL/6J mice, scale bar 5 µm. (**C**) Quantitative PCR mRNA expression levels of Hv1 in the C57BL/6J mouse striatum following sub-chronic LPS treatment. (**D**) Representative immunofluorescent images of Hv1 protein levels in Iba1-positive cells in the SN of C57BL/6J mice following saline or LPS treatment, scale bar 5 µm. (**E**,**F**) C57BL/6J primary microglia treated with LPS increase *Hv1* mRNA and protein levels, measured by qPCR (**E**) and quantified immunofluorescent images (**F**); scale bar 20 µm. *n* = 4–5 mice per group and 3 isolations for primary microglia. Asterisks denote statistically significant differences—* *p* < 0.05, ** *p* < 0.01, *** *p* < 0.001, using unpaired Student’s *t*-test. Error bars denote the standard error of the mean.

Next, we utilized the well-characterized acute MPTP mouse model of PD to examine *Hvcn1* expression. MPTP readily penetrates the blood–brain barrier and rapidly oxidizes to MPP+, which is toxic to mitochondria and affects the electron transport chain, thereby increasing ROS generation [59,60]. MPP+ is also taken up by the dopamine transporter and is concentrated in the dopaminergic neurons, leading to the loss of nigrostriatal neurons, a hallmark of PD pathology and Parkinsonism [61,62,63]. MPTP (10 mg/kg) was injected once every two hours for four total injections, and the mice were sacrificed either 2 or 7 days later [64,65]. This paradigm is known to induce an inflammatory response and the loss of dopaminergic neurons in the SN [66]. We found a significant 2-fold increase in Hvcn1 mRNA expression in the striatum (Figure 2A) and visibly increased Hv1 protein levels by immunofluorescence microscopy in the SN (Figure 2B) two days following MPTP treatment. In addition, we used a sub-chronic LPS injection paradigm (1 mg/kg once daily for four consecutive days) and collected the tissue 24 h after the last injection. The LPS injection paradigm led to ~6-fold higher levels of Hvcn1 gene expression in the striatum compared to saline-treated animals (Figure 2C). Further, immunofluorescent microscopy confirmed that Hv1 protein was colocalized with Iba1-positive cells in the mouse SN and dramatically increased with LPS treatment (Figure 2D). Cultured C57BL/6J primary microglia basally express Hv1, and treatment with LPS (100 ng/mL) for 6 h induced a statistically significant 2-fold increase in Hvcn1 gene expression (Figure 2E), along with a substantial increase in Hv1 protein level visualized by immunofluorescent images (Figure 2F, left panel) and quantified immunofluorescent intensity (Figure 2F, right panel).

### 3.2. Hv1 KO Mice Have Lower Basal Inflammatory Status and Exhibit a Reduced MPTP-Mediated Inflammatory Response in the Mouse Nigrostriatal Pathway

**Figure 3:**
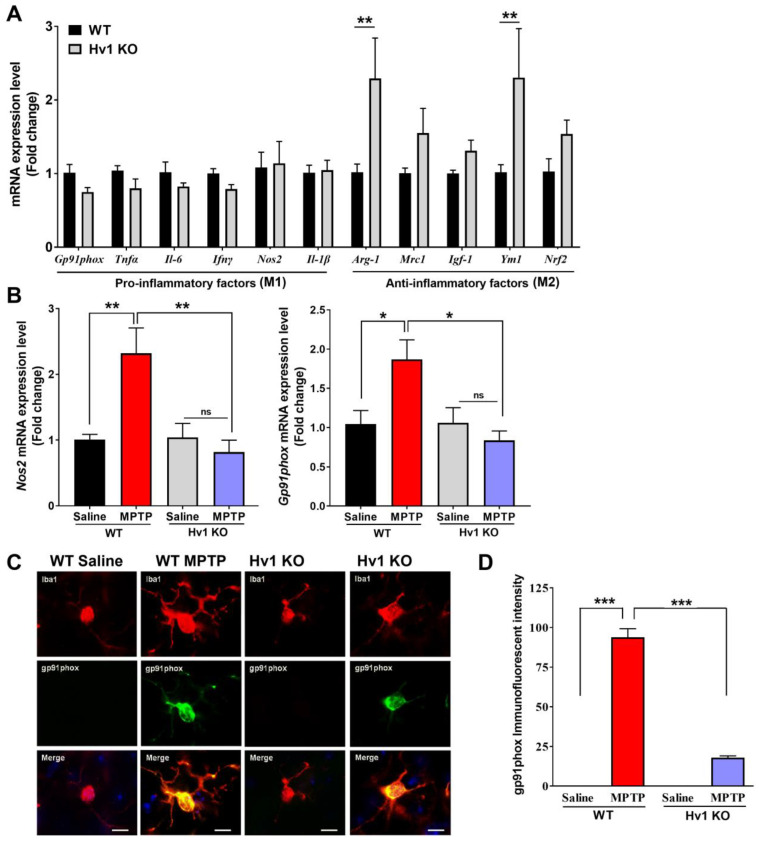
Basal inflammatory status and MPTP-induced ROS and NO are reduced in Hv1 KO mice. (**A**) Quantitative PCR results for basal levels of pro-inflammatory factors (*Gp91phox*, *Tnfα*, *Il-6*, *Ifnγ*, *Nos2*, and *Il-1β*) and anti-inflammatory factors (*Arginase-1*, *Mrc1*, *Igf-1*, *Ym1*, and *Nrf2*) in the striatum of Hv1 KO mice compared to WT mice. ** *p* < 0.01 using SEM by Student’s *t*-test. (**B**) Quantitative PCR results for the genes *Nos2* (left) and *Gp91phox* (right) in the WT and Hv1 KO mouse striatum 2 days after MPTP treatment with MPTP treatment compared to saline for each genotype. (**C**) Representative immunofluorescent images of gp91phox (green) in IBA1-positive (red) cells, with DAPI nuclei stain (blue) in WT and Hv1 KO sections treated with saline or MPTP, scale bar 5 μm. (**D**) Semi-quantification of Gp91phox protein fluorescent intensity levels. *n* = 4–5 mice per group for all experiments. Asterisks denote statistically significant differences—* *p* < 0.05, ** *p* < 0.01, *** *p* < 0.001, using two-way ANOVA followed by Tukey’s multiple comparisons. ‘ns’ indicates not statistically significant. Error bars denote the standard error of the mean.

Because Hv1 is mainly expressed in microglia, we investigated the inflammatory status of Hv1 KO striatum compared to WT mice under basal conditions. We found that Hv1 KO mice had reduced basal gene expression of pro-inflammatory factors (*Gp91phox*, *Tnfα*, *Il-6*, *Ifnγ*, *Nos2*, and *Il-1β*), with increased gene expression of each anti-inflammatory factor measured (*Arginase-1*, *Mrc1*, *Igf-1, Ym1,* and *Nrf2)* (Figure 3A). The significant increase in *Arginase-1* and *Ym1* expression indicates that Hv1 KO mice may have higher basal anti-inflammatory alternatively activated microglia, also termed M2, in the striatum. Next, we determined whether Hv1 KO could alter the MPTP-induced generation of ROS and nitric oxide (NO). We measured the mRNA levels of iNOS (encoded by the gene *Nos2*), the enzyme responsible for generating NO, and *Gp91phox*, a component of the NADPH oxidase system responsible for generating ROS. MPTP treatment in WT mice significantly increased the mRNA levels of these two genes by 2-fold compared to controls. Hv1 deficiency completely blocked this MPTP-induced upregulation (Figure 3B). We further utilized immunofluorescent imaging to measure the protein level of the active component of the NADPH oxidase system, gp91phox (NOX2), in Iba1-positive cells. Similarly, we found that Hv1 KO mice treated with MPTP have a 75-fold reduction in the protein level of gp91phox compared to MPTP-treated WT mice, which expressed over a 90-fold increase compared to WT saline animals (Figure 3C,D).

**Figure 4:**
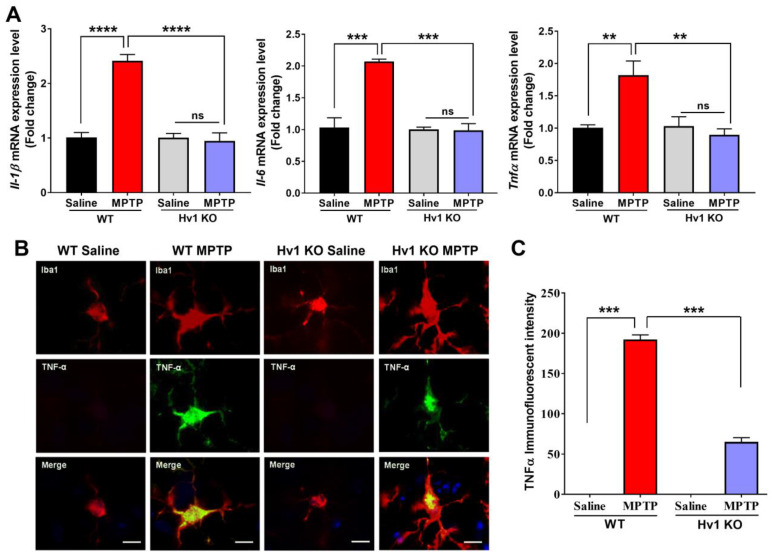
Hv1 KO mice exhibit attenuated MPTP-induced pro-inflammatory cytokine production. (**A**) Quantitative PCR results demonstrate that Hv1 KO mice completely abolish the MPTP-induced mRNA levels of the inflammatory cytokines *Il-1b* (**left**), *Il-6* (**middle**), and *Tnfa* (**right**) compared to WT mice 2 days after MPTP treatment. (**B**) Representative immunofluorescent images of TNFα (green) in IBA1-positive (red) cells, with DAPI nuclei stain (blue) in WT and Hv1 KO sections treated with saline or MPTP, scale bar 5 μm. (**C**) Semi-quantification of TNFα protein fluorescent intensity levels. *n* = 4–5 mice per group for all experiments. Asterisks denote statistically significant differences, ** *p* < 0.01, *** *p* < 0.001, and **** *p* < 0.0001, using two-way ANOVA followed by Tukey’s multiple comparisons. ‘ns’ indicates not statistically significant. Error bars denote the standard error of the mean.

Next, we measured the levels of pro-inflammatory cytokines in the MPTP-treated WT and Hv1 KO mice two days after MPTP injections. Gene expression levels of *Il-1b*, *Il-6*, and *Tnfa* were significantly increased in MPTP-treated WT striatum, whereas Hv1 KO mice expressed control levels of these inflammatory cytokines (Figure 4A). To confirm our gene expression results, we used immunofluorescent imaging to visualize the protein level of the pro-inflammatory cytokine TNFα (Figure 4B). WT mice treated with MPTP demonstrated over 190-fold induction of TNFα protein levels, whereas in Hv1 KO mice, this increase was significantly attenuated, with a 65-fold increase, much less than that in the WT saline group (Figure 4C). Taken together, these data demonstrate that mice lacking Hv1 have attenuated basal inflammatory cytokine production and an overall inflammatory phenotype, along with a reduced inflammatory response to the neurotoxin MPTP.

### 3.3. Hv1 KO Mice Are Protected from MPTP-Induced Dopaminergic Neurotoxicity

**Figure 5:**
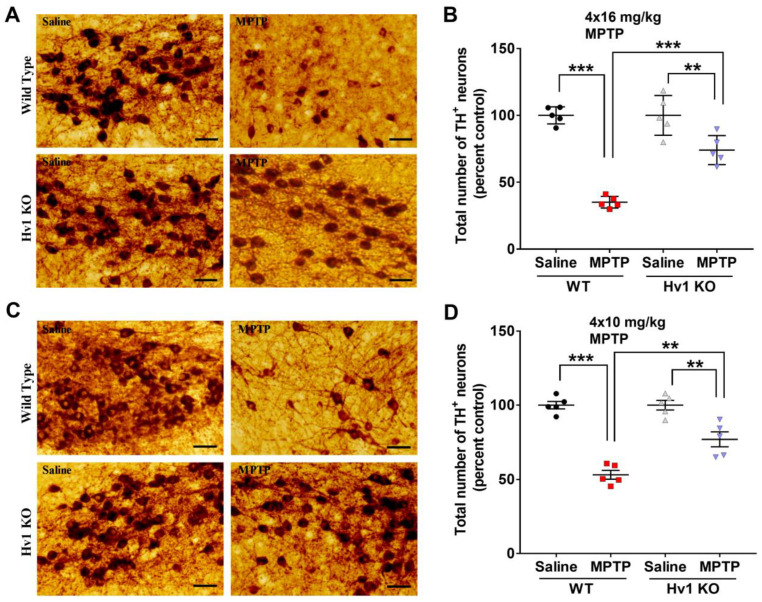
Hv1 deficient mice have diminished MPTP-mediated loss in TH-positive neurons in the substantia nigra. (**A**,**C**) Representative 20× DAB immunostaining images for TH in the SN of WT and Hv1 KO mice following acute 4 × 16 mg/kg (**A**) or 4 × 10 mg/kg (**C**) MPTP treatment, scale bar 50 µm. (**B**,**D**) Stereological counts for the total number of TH-positive neurons in the SN following 4 × 16 mg/kg (**B**) or 4 × 10 mg/kg (**D**) in each genotype. *n* = 5 mice per group. Asterisks denote statistically significant differences—** *p* < 0.01, *** *p* < 0.001, using two-way ANOVA followed by Tukey’s multiple comparisons. ‘ns’ indicates not statistically significant. Error bars denote the standard error of the mean.

The pathogenesis of PD revolves around the loss of dopaminergic neurons in the SN region of the midbrain [1,67]. We found an average TH^+^ cell loss of 65% in WT mice treated with MPTP (4 × 16 mg/kg), whereas global Hv1 KO mice demonstrated only an average of 39% loss (Figure 5A,B) when compared to the WT saline group. To investigate whether reducing the MPTP dose could lead to increased protection in the Hv1 KO mice, we employed an acute 4 × 10 mg/kg MPTP treatment. The lower dose induced an average of 47% loss of TH-positive cells in saline-treated WT mice, whereas the same treatment resulted in a 28% loss in the Hv1 KO mice when compared to saline-treated WT animals (Figure 5C,D). Therefore, Hv1 deficiency significantly attenuated the MPTP-induced loss of TH-positive neurons, with similar protection at two different doses of MPTP.

### 3.4. Hv1 KO Mice Are Protected from LPS-Induced Neuroinflammation and Dopaminergic Neurotoxicity

**Figure 6:**
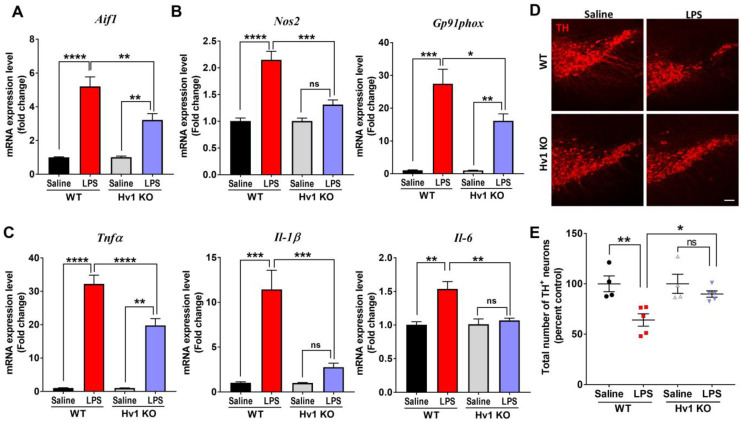
Hv1 KO mice exhibit reduced inflammatory response and protection of TH-positive neurons in an LPS model of PD. (**A**–**C**) Quantitative PCR mRNA levels of inflammatory factors in the striatum of WT or Hv1 KO mice following sub-chronic LPS treatment, including mRNA levels of *Aif1* (**A**), *Nos2,* and *Gp91phox* (**B**), and pro-inflammatory cytokines *Tnfα*, *Il-1β,* and *Il-6* (**C**). (**D**) Representative 10× immunofluorescent images for TH-positive neurons in the SN following treatment of LPS in WT and Hv1 KO mice, scale bar 100 µm. (**E**) Stereological counts for a total number of TH-positive neurons in the SN following subchronic LPS treatment in each genotype. *n* = 4–5 mice per group for all experiments. Asterisks denote statistically significant differences—* *p* < 0.05, ** *p* < 0.01, *** *p* < 0.001, and **** *p* < 0.0001, using two-way ANOVA followed by Tukey’s multiple comparisons. ‘ns’ indicates not statistically significant. Error bars denote the standard error of the mean.

MPTP is a selective dopaminergic neurotoxin that induces an inflammatory response by damaging neurons. Therefore, we wanted to further characterize the role of Hv1 in a direct inflammatory toxicant animal model of PD utilizing LPS, a purified component of the Gram-negative bacterial cell wall, as a direct inflammogen. LPS injection (1 mg/kg) given once daily to mice for four straight days and animals sacrificed one day after the last injection, induced striatal gene expression of the microglial marker *Aif1*, the gene name for Iba1, by 5-fold in WT mice compared to saline-treated WT mice (Figure 6A), analogous to our MPTP findings. Compared to the WT LPS group, Hv1 KO mice showed significantly reduced LPS-induced *Aif1* (Iba1) mRNA upregulation, indicating a potential reduction in microglial proliferation and inflammatory activation. Furthermore, the inflammatory genes *Nos2* and *Gp91phox* were significantly increased in WT mice following LPS treatment, and Hv1 KO mice exhibited significantly attenuated upregulation of these same genes (Figure 6B). Because the LPS-mediated inflammatory response leads to increased production of pro-inflammatory cytokines, we examined whether cytokine mRNA expression was induced in Hv1 KO mice. LPS treatment in WT mice led to a significant increase in the gene expression of all three measured cytokines, and Hv1 KO mice expressed significantly lower levels of *Tnfa*, *Il-1b*, and *Il-6* with the same treatment (Figure 6C).

Repeated LPS injections lead to the loss of dopaminergic neurons in the SN [46,68]. As such, we determined whether Hv1 deficiency could ameliorate LPS-mediated neurotoxicity resulting from the high- and low-dose acute MPTP treatment paradigms. LPS treatment in WT mice induced an average 36% loss in TH^+^ cells. However, the same treatment paradigm in Hv1 KO mice resulted in an average of 12% TH^+^ cell loss, which was not statistically significant from saline-treated WT or Hv1 KO mice (Figure 6D,E). Notably, Hv1 KO mice did not exhibit a basal reduction in total TH^+^ neurons compared to WT animals, indicating that deficiency of Hv1 does not lead to any measurable neurotoxicity under control conditions. These data suggest that the lack of Hv1 can attenuate the neuroinflammation and dopaminergic neurodegeneration induced by a direct inflammatory toxicant.

### 3.5. Hv1 KO Attenuates LPS-Induced Reactive Oxygen and Nitrogen Species Generation in Primary Mouse Microglia

**Figure 7:**
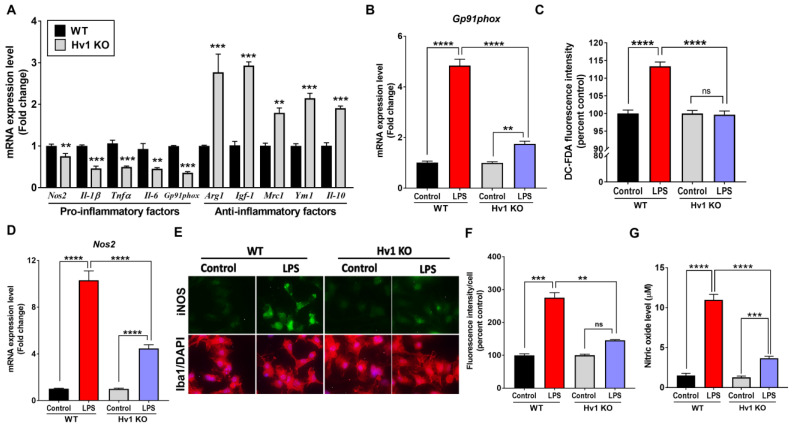
Hv1 KO attenuates LPS-induced reactive oxygen and nitrogen species generation in primary mouse microglia. (**A**) Basal gene expression of M1- or M2-related factors in WT versus Hv1 KO primary microglia via qPCR. ** *p* < 0.01, and *** *p* < 0.001 using SEM by Student’s *t*-test. (**B**,**C**) Hv1 KO primary microglia have reduced ROS generation following LPS treatment compared to WT control microglia, measured by *Gp91phox* gene expression (**B**) and intracellular ROS generation measured by the fluorescent CM-H_2_DCFDA dye (**C**). Hv1 KO primary microglia have reduced *Nos2* gene expression (**D**) following LPS treatment compared to WT control microglia treated with LPS. Representative immunofluorescent images (**E**) for iNOS (green) in Iba1-positive (red) cells following LPS treatment in WT and Hv1 KO primary microglia, along with DAPI nuclear stain (blue) (scale bar 20 μm), with iNOS fluorescent intensity quantification (**F**), and media nitrite levels (**G**) measured by the colorimetric Griess assay. *n* = 3 isolations for all experiments. Asterisks denote statistically significant differences, ** *p* < 0.01, *** *p* < 0.001, and **** *p* < 0.0001, using two-way ANOVA followed by Tukey’s multiple comparisons. ‘ns’ indicates not statistically significant. Error bars denote the standard error of the mean.

We utilized purified, isolated primary postnatal (0–3 days old) mouse microglia to determine the expression and function of Hv1 in these isolated cells. A recent study showed that primary microglia isolated from Hv1 KO mice have a higher expression of the M2 factors Arginase-1 and Mrc1 when treated with IL-4 and reduced expression of M1 factors iNOS and CD16 when treated with LPS and IFNγ [69]. We further determined whether Hv1 deficiency could protect dopaminergic neurons from the activated microglia inflammatory response. After confirmation that cultured microglia isolated from Hv1 KO mice were, in fact, Hv1 deficient via qPCR and fluorescent microscopy (Supplemental Figure S1), we characterized the basal gene expression of both pro- (M1) and anti-inflammatory (M2) factors in these cells compared to WT microglial cultures. Similar to our observations in Hv1 KO animals, Hv1 KO microglia exhibited significantly reduced basal gene expression of all pro-inflammatory factors measured (*Gp91phox*, *Tnfα*, *Il-6*, *Ifnγ*, *Nos2*, and *Il-1β*), accompanied by significantly increased gene expression of each anti-inflammatory factor (*Arginase-1*, *Mrc1*, *Igf-1*, *Ym1*, and *Nrf2)* (Figure 7A).

Because Hv1 is linked to ROS production via activation of the NADPH oxidase system [29], including the active subunit gp91phox, we initially measured this pathway to establish whether microglia lacking Hv1 would have an attenuated ROS response to LPS. LPS-treated microglia isolated from WT mice exhibited a significant increase in both *Gp91phox* gene expression (Figure 7B) and intracellular reactive oxygen species (iROS) generation, as measured by the fluorescent dye H_2_DC-FDA (Figure 7C). Hv1 KO primary microglia demonstrated significantly lower mRNA levels of *Gp91phox* and iNOS when treated with LPS compared to WT cultures. As the in vivo results showed reduced iNOS protein levels due to Hv1 deficiency, we measured *Nos2* gene expression, protein level, and nitric oxide generation. LPS treatment induced a 10-fold increase in *Nos2* gene expression (Figure 7D) and an almost 3-fold increase in iNOS protein levels (Figure 7E,F) in WT microglia. In contrast, Hv1 KO microglia produced significantly less iNOS mRNA and protein, with a 4- and 1.5-fold induction, respectively. Griess assay was used to determine media nitrite levels following LPS treatment. We observed that WT microglia treated with LPS produced a 4-fold increase in media nitrite levels, and Hv1 KO microglia significantly attenuated this response (Figure 7G). Together, these data validate that Hv1 deficiency reduces NO production and significantly attenuates LPS-induced iNOS upregulation.

### 3.6. Hv1 KO Attenuates LPS-Induced Inflammatory Cytokine Production and Blocks the Reduction of Anti-Inflammatory Factors in Primary Mouse Microglia

**Figure 8:**
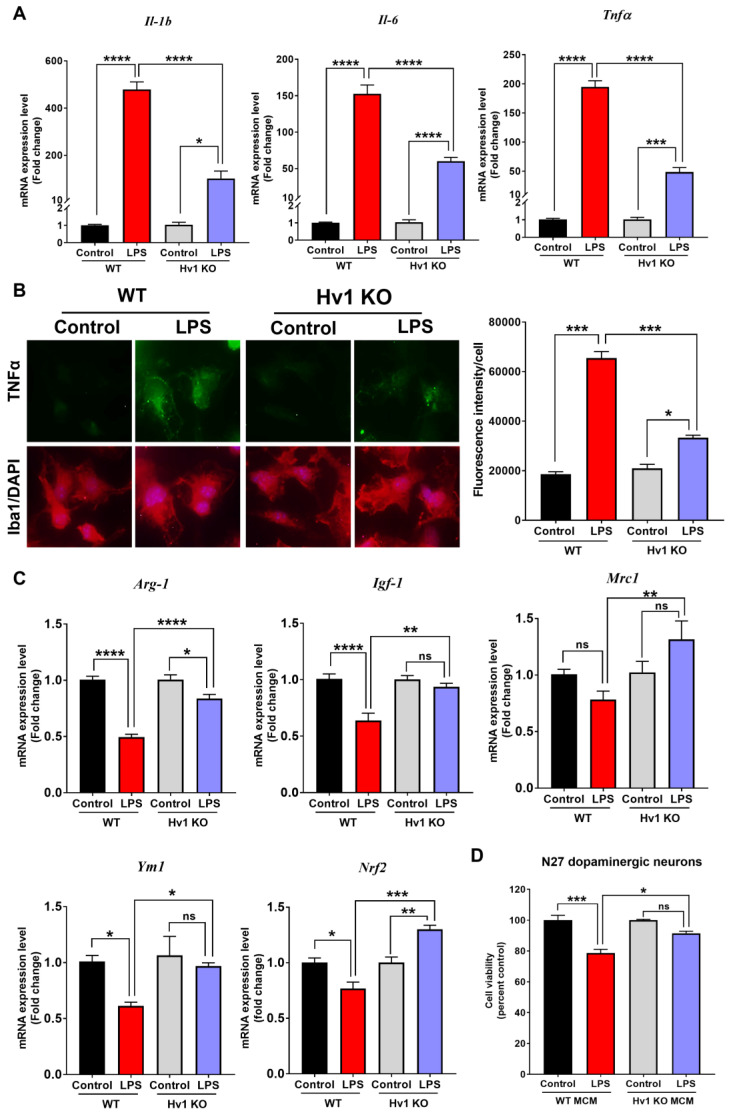
Hv1 KO attenuates LPS-induced inflammatory response in primary mouse microglia and protects dopaminergic neurons from LPS-treated microglial conditioned media. (**A**) Primary microglia isolated from Hv1 KO mouse pups have reduced Hv1 mRNA levels of the inflammatory cytokines *Il-1b*, *Il-6*, and *Tnfa* by qPCR compared to LPS-treated WT microglia. (**B**) Representative immunofluorescence images for TNFα (green) in Iba1-positive cells (red), with nuclear DAPI stain (blue), scale bar 20 μm. TNFα fluorescence quantification (right). (**C**) Quantitative PCR gene expression results for the anti-inflammatory genes *Arginase-1*, *Igf-1*, *Mrc1*, *Ym1*, and *Nrf2* in WT and Hv1 KO primary microglia following LPS treatment. (**D**) Cell viability of N27 rat dopaminergic neurons was measured using MTS reagent that produces a formazan product in metabolically active cells. Media conditioned from primary microglia (microglia-conditioned media or MCM) treated with or without LPS for 24 h was added to N27 cells for 24 h. *n* = 3 isolations for primary microglia and 3 separate passages for N27 cells. Asterisks denote statistically significant differences—* *p* < 0.05, ** *p* < 0.01, *** *p* < 0.001, and **** *p* < 0.0001, using two-way ANOVA followed by Tukey’s multiple comparisons. ‘ns’ indicates not statistically significant. Error bars denote the standard error of the mean.

Although it has previously been reported that Hv1 regulates iNOS and CD16 protein levels in cultured microglia [69], the role of Hv1 in microglial cytokine production has not been examined. After demonstrating reduced basal gene expression of several pro-inflammatory cytokines in vivo and in cultured microglia (Figure 3A and Figure 7A), we examined whether Hv1 deficiency could attenuate LPS-induced cytokine production. Resembling the basal gene expression data, LPS-treated Hv1 KO microglia expressed significantly less *Il-1b*, *Il-6*, and *Tnfa* mRNA compared to LPS-treated WT microglia (Figure 8A). To further assess the effect of LPS, we used immunocytochemistry and fluorescent microscopy to determine the protein level of TNFα in wild-type and Hv1 KO cultured microglia. Again, Hv1 KO microglia had significantly lower protein levels of TNFα following LPS treatment compared to WT microglia treated with LPS (Figure 8B).

IL-4 treatment in Hv1 KO microglia increases arginase-1 protein levels [69]. Therefore, we measured the mRNA levels of multiple anti-inflammatory M2-related genes in Hv1 KO microglia post-LPS treatment, including *Arginase-1*. After 6 hr LPS treatment of WT microglia, the gene expression of *Arginase-1*, *Igf-1*, *Ym1*, and *Nrf2* was significantly reduced, with a reduction in *Mrc1* as well (Figure 8C). However, Hv1-deficient microglia demonstrated an attenuated response to the LPS-induced reduction for each of the M2-related factors measured herein. In addition, *Mrc1* and *Nrf2* revealed an increase in gene expression after LPS treatment. These results demonstrate that Hv1 deficiency in cultured microglia protects against the LPS-induced reduction of anti-inflammatory factors.

### 3.7. Hv1 Deficiency in Microglia Protects Dopaminergic Neuron Viability from LPS-Mediated Microglial Inflammatory Response

To determine whether the reduced LPS-mediated inflammatory response in Hv1 KO primary microglia resulted in direct neuroprotection, we treated the rat N27 dopaminergic neuronal cell line with microglia-conditioned media (MCM) from WT or Hv1 KO cultures treated with or without LPS. N27 cells were treated with 250 nM rotenone for 24 h as a positive control to induce at least 20% loss of viability, as determined by the MTS assay (Supplemental Figure S2). MCM from WT cultures treated with LPS for 24 h resulted in about 25% loss of N27 cell viability, significantly less than control WT MCM (Figure 8D). MCM from Hv1 KO cultures treated with LPS caused less than 10% loss of N27 cell viability, with significantly higher cell viability when compared to WT LPS MCM-treated N27 cells. Therefore, the reduced LPS-mediated inflammatory response in Hv1-deficient microglia results in an attenuated loss of dopaminergic neuronal viability.

## 4. Discussion

Parkinson’s disease (PD) is a debilitating neurodegenerative disease with no current disease-modifying therapies available. Microglial activation and neuroinflammation are hallmarks of PD and play an essential role in disease progression [5,68,70,71,72,73]. The molecular mechanisms that drive the microglial inflammatory response are still being elucidated, although, growing evidence indicates that ion channels are involved in the pathogenesis of neurodegenerative diseases, including PD, and may serve as therapeutic targets [74,75]. Here, we report that substantia nigra samples from post-mortem PD patients express higher mRNA levels of *HVCN1* compared to age-matched controls, with PD males expressing 30% more *HVCN1* compared to age-matched controls and no difference in female PD patients compared to controls. These data are in parallel with the motor deficits and development of PD pathophysiology observed earlier and at a greater level in males than females [43,76]. Males also show an increased sensitivity of microglia to an inflammatory insult, albeit dependent on other factors, including age, brain region, and environment [77]. A recent transcriptomic study found differentially expressed genes in male and female PD patients [78]. Current literature also suggests that estrogen in females may play a significant role in the trajectory and extent of developing PD pathology, including the ability to attenuate microglial activation [43,79,80]. These sex-based differences may explain the sex-specific observations in upstream Hv1 levels. However, further experiments corroborating this finding need to be carried out. These data indicate that Hv1 is expressed significantly higher in the substantia nigra of PD patients compared to control patients, and understanding the molecular pathways and mechanisms that drive PD pathogenesis could elucidate novel therapeutic targets to halt disease progression.

Similar to the human expression data, a significant increase in *Hvcn1* mRNA level was observed in the striatum two days following the last injection of MPTP, which is consistent with findings that 24–48 h post-MPTP represents the peak inflammatory response in the striatum [81,82] and precedes dopamine neuron loss in this PD model [83]. Increased Hvcn1 gene expression was also observed one day after the last injection in a subchronic LPS model of PD that resulted in a dramatic microglial activation phenotype [46]. This is the first study to examine the expression of Hv1 and Hv1-regulated neuroinflammation in animal models of Parkinson’s disease. The data presented here indicate that Hv1 expression could represent an early indicator or response to damage in the nigrostriatal system.

Our findings, along with others, indicate that Hv1 is specifically localized to microglia in the brain [29,34]. Since striatum *Hvcn1* expression is increased following a neurotoxic stimulus, we hypothesized that Hv1 expression might mediate the inflammatory status of the microglia. In support of this hypothesis, global Hv1 KO mice have a trend towards reduced basal production of M1-related inflammatory factors (*Nos2*, *gp91phox*, *Tnfα*, *Il-1β*, *Il-6*, and *Ifnγ*) in the striatum, but none of the pro-inflammatory genes measured demonstrated a statistically significant reduction compared to WT mice. However, Hv1 KO mice express significantly higher M2-related anti-inflammatory factors *Arginase-1* and *Ym1*, with trends of increasing *Igf-1*, *Mrc1*, and *Nrf2*. This is particularly important, as we have recently demonstrated that alternative M2 activation is associated with the cessation of progressive dopamine neuron loss following systemic LPS exposure [46]. The effect of Hv1 KO on the basal inflammatory signal from mouse brain tissue is dampened, as other cells also express these inflammatory factors, and Hv1 is specifically expressed in microglia. We note that the basal expression of Iba1 (encoded by the gene *Aif1*), along with observable numbers of Iba1-positive cells, does not differ between WT and Hv1 KO mice. Along with reducing the basal inflammatory status, Hv1 deficiency completely abolished the inflammatory response in the striatum from the selective dopaminergic neurotoxicant MPTP and the inflammogen LPS. This effect includes decreased mRNA levels of factors responsible for the generation of nitric oxide (*Nos2*) and ROS (*Gp91phox*), along with pro-inflammatory cytokines *Il-1b*, *Il-6*, and *Tnfa*, which consequently reduced protein levels of both gp91phox and TNFα. Further, after MPTP treatment, gp91phox and TNFα protein levels in Iba1-positive microglia were significantly attenuated in Hv1 KO mice. Together, these data demonstrate that Hv1 is involved in the basal inflammatory status in the mouse brain and that a deficiency in Hv1 can reduce the MPTP and LPS-mediated inflammatory response, specifically in microglia.

Oxidative stress is another factor implicated in the progression of PD, and decreasing the production of reactive molecules by inhibition of NADPH oxidase leads to increased neuroprotection [84]. Hv1 regulates NADPH oxidase activity and reduces the production of ROS in an animal model of ischemia, along with the findings that Hv1 KO microglia both in vitro and in vivo demonstrate reduced protein levels of the pro-inflammatory factors iNOS and CD16 following LPS plus interferon-γ treatment or during ischemic injury, respectively [69]. Therefore, we postulated that Hv1 deficiency leads to alterations in factors associated with ROS and NO production. We show that Hv1 KO mice exhibit lower mRNA levels of *Nos2* in the striatum both basally and following MPTP or LPS treatment compared to WT animals. Similarly, isolated Hv1-deficient microglia in culture express significantly less *Nos2* both basally and following LPS treatment. We measured the mRNA and protein levels of the active subunit of NADPH oxidase, *Gp91phox*, along with the measurement of ROS generation in cultured Hv1 KO microglia. As we anticipated, Hv1-deficient microglia expressed lower *Gp91phox* mRNA levels along with reduced ROS generation following LPS treatment compared to WT microglia. Evidence from previous studies indicates that deficiency of Hv1 results in the reduction of ROS in other cell types, including B-cells [85], bone marrow cells [36], and neutrophils [86]. Consistent with previous findings, albeit in other ROS-producing cells, in this study, we show that Hv1 KO microglia produce less iNOS protein, indicating that Hv1 deficiency leads to reduced levels of the damaging factors NO and ROS. Importantly, cells from Hv1 KO mice attenuate but do not abolish ROS levels, which is essential since a lack of ROS can impair several metabolic and developmental pathways [87], thus favoring the therapeutic potential of inhibiting Hv1 [88,89].

Although Hv1 regulates the production of ROS, the modulation of pro-inflammatory cytokines in microglia by Hv1 has not been investigated, so we sought to characterize this aspect of the microglial inflammatory response because of its importance in PD and as a potential mechanism for neuronal toxicity. Similar to findings in human PD patients and animal models of PD, LPS significantly increased Hv1 gene expression, suggesting that the LPS-mediated increased Hv1 may be involved in the promotion of inflammation as part of the M1 microglial pro-inflammatory activation phenotype. Further support for this hypothesis is provided by the observation that Hv1 KO microglia have significantly reduced basal production of M1-related inflammatory cytokines (*Tnfa, Il-1b*, and *Il-6*) while significantly increased M2-related anti-inflammatory factors (*Arginase-1*, *Igf-1*, *Mrc1*, *Ym1*, and *Nrf2*) compared to WT microglia. Additionally, Hv1-deficient microglia significantly attenuate the inflammatory response to LPS compared to WT cultures, with diminished gene expression of the pro-inflammatory cytokines *Il-1b*, *Il-6,* and *Tnfa*, along with reduced protein levels of TNFα. On the other hand, Hv1 deficiency also abolishes the LPS-mediated reduction of gene expression for the M2-related anti-inflammatory factors *Arginase-1*, *Igf-1*, *Mrc1*, *Ym-1,* and *Nrf2*. Tian et al. found that Hv1-deficient microglia produced higher levels of arginase-1 and mrc1 following treatment with the anti-inflammatory cytokine IL-4 [69]. However, our study is the first to demonstrate that Hv1 KO microglia dampen the LPS-mediated reduction of multiple M2-related factors. These data further support a role for Hv1 deficiency in shifting the inflammatory status of cultured microglia towards an anti-inflammatory phenotype, with increased expression of inflammation-resolving factors. This anti-inflammatory role of Hv1 deficiency is most likely linked to the regulation of NADPH oxidase activity since this enzyme has been linked to NFκB activation along with the generation of ROS and will lead to downstream inflammatory cytokine production [90]. Therefore, Hv1 could play a crucial role in mediating the microglial M1 (pro-inflammatory) and M2 (anti-inflammatory) phenotypes.

Anti-inflammatory therapies for PD have produced mixed results [91,92]. There are many factors that can reduce the inflammatory response, but reduced inflammation does not significantly alter the disease course. Therefore, we investigated whether the reduced inflammatory response in Hv1 KO microglia could lead to the direct protection of dopaminergic neurons. We found that lack of Hv1 significantly protects the total population of TH^+^ neurons from two different MPTP treatment paradigms, along with protection against LPS-induced neurodegeneration. To confirm that this protection is mediated through reduced microglial inflammation, we performed conditioned media experiments on cultured microglia and neurons. The microglial conditioned media (MCM) from WT microglia treated with LPS resulted in a significant loss of cell viability that was attenuated in Hv1 KO microglia, similar to that found in an animal model of ischemia, with Hv1 deficiency leading to neuroprotection [34,69]. Taken together, these data indicate that the Hv1-mediated inflammatory response in microglia can directly lead to dopaminergic neurotoxicity and that removal of Hv1 significantly abolishes this effect. These results, paired with the human expression data demonstrating increased Hv1 expression in PD patients, reveal that Hv1 is a potential therapeutic option for PD to reduce neuroinflammation and dopaminergic neurotoxicity.

## 5. Conclusions

In summary, we characterize a novel mechanism for regulating chronic inflammation in PD. Our data show that the voltage-gated proton channel Hv1 plays an essential role in regulating the microglial inflammatory response in PD models. We also establish that post-mortem PD patients and animal models of PD, along with cultured primary microglia challenged with LPS, express higher levels of Hv1. Moreover, Hv1-deficient mice treated with MPTP or LPS showed a dampened inflammatory response compared to WT mice, which leads to significant dopaminergic neuroprotection in both neurotoxicant models. Further characterization in a cell culture model shows that Hv1 deficient microglia produce less pro-inflammatory factors following LPS exposure, which results in significantly increased dopaminergic neuron viability compared to LPS-challenged WT microglia. Collectively, our study elucidates a novel role for Hv1 in sustained microglia-mediated neuroinflammation in dopaminergic neurodegeneration observed in PD.

## Data Availability

The data presented in this study are available in the article and supplementary materials.

## Supplementary Materials

The following supporting information can be downloaded at: https://www.mdpi.com/article/10.3390/antiox12030582/s1, Figure S1: Confirmation of Hv1 knockout in primary microglia. Asterisks denote statistically significant differences—*** *p* < 0.001; Figure S2: Rotenone induced loss of N27 cell viability. As-terisks denote statistically significant differences—** *p* < 0.01; Table S1:qPCR primer list.
