# Supplementary material for: Voltage-Gated Proton Channel Hv1 Regulates Neuroinflammation and Dopaminergic Neurodegeneration in Parkinson’s Disease Models
Source: Antioxidants (Basel). 2023 Feb 25;12(3):582. doi: 10.3390/antiox12030582 (PMC10044828; doi:10.3390/antiox12030582)
Supplement: Supplementary file 1 [file antioxidants-12-00582-s001.zip › Supplemental Table S1.pdf]

Gene	Forward	Reverse	Accession Number
Hvcn1	TGCAAAGGAGTGCTGCAAACTA	TCGAGTAGACGCTCCGCAAT	NM_001359454
Gp91phox	CCAACTGGGATAACGAGTTCA	GAGAGTTTCAGCCAAGGCTTC	NM_007807
Nos2	TCACGCTTGGGTCTTGTT	CAGGTCACTTTGGTAGGATTT	NM_010927
Il-6	TCCATCCAGTTGCCTTCTTG	ATTGCCATTGCACAACTCTTTT	NM_001314054
Tnfα	AGGGATGAGAAGTTCCCAAATG	TGTGAGGGTCTGGGCCATA	NM_001278601
Il-1β	AGTTGACGGACCCCAAAAGAT	GGACAGCCCAGGTCAAAGG	NM_008361
Ifnγ	ATGAAAATCCTGCAGAGCCA	GTGGGTTGTTGACCTCAAACT	NM_008337
Arginase-1	GGACCTGGCCTTTGTTGATG	AGACCGTGGGTTCTTCACAATT	NM_007482
Igf-1	CGCCTCATTATCCCTGCCCACCA	GCCATAGCCTGTGGGCTTGTTGAA	NM_010512
Mrc1	CCCAAGGGCTCTTCTAAAGCA	CGCCGGCACCTATCACA	NM_008625
Ym1	TCTGGTGAAGGAAATGCGTAAA	GCAGCCTTGGAATGTCTTTCTC	NM_009892
Nrf2	CTCGCTGGAAAAAGAAGTG	CCGTCCAGGAGTTCAGAGG	NM_010902
Aif1	CAGACTGCCAGCCTAAGACA	AGGAATTGCTTGTTGATCCC	NM_001361501
Gapdh	TGAAGCAGGCATCTGAGGG	CGAAGGTGGAAGAGTGGGAG	NM_001289726
Rpl13a	CTGTGAAGGCATCAACATTTCTG	GACCACCATCCGCTTTTTCTT	NM_009438
