# Supplementary figures and images for: Voltage-Gated Proton Channel Hv1 Regulates Neuroinflammation and Dopaminergic Neurodegeneration in Parkinson’s Disease Models
Source: Antioxidants (Basel). 2023 Feb 25;12(3):582. doi: 10.3390/antiox12030582 (PMC10044828; doi:10.3390/antiox12030582)

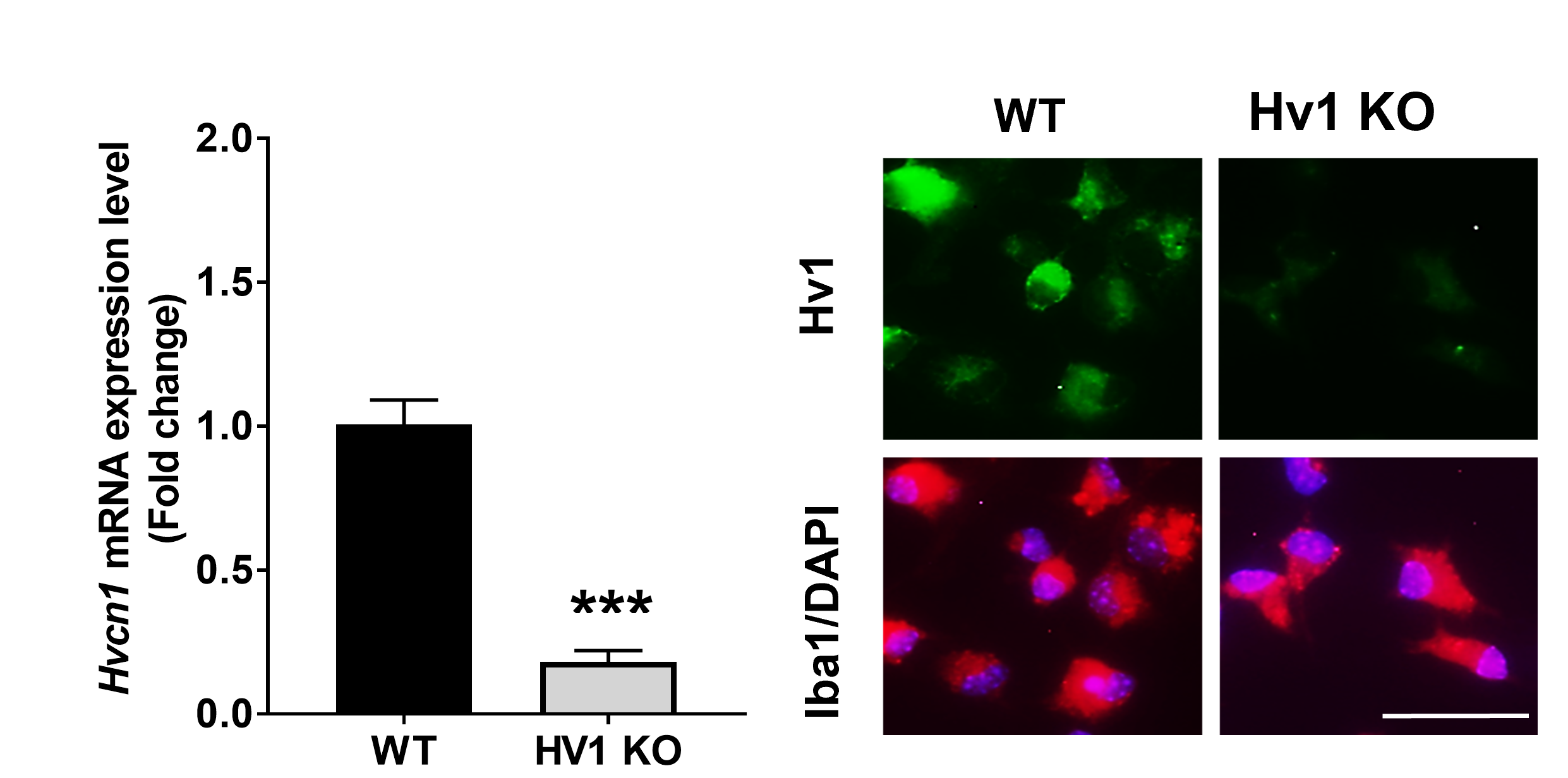

Supplement: Supplementary file 1 [file antioxidants-12-00582-s001.zip › Figure S1.tif]

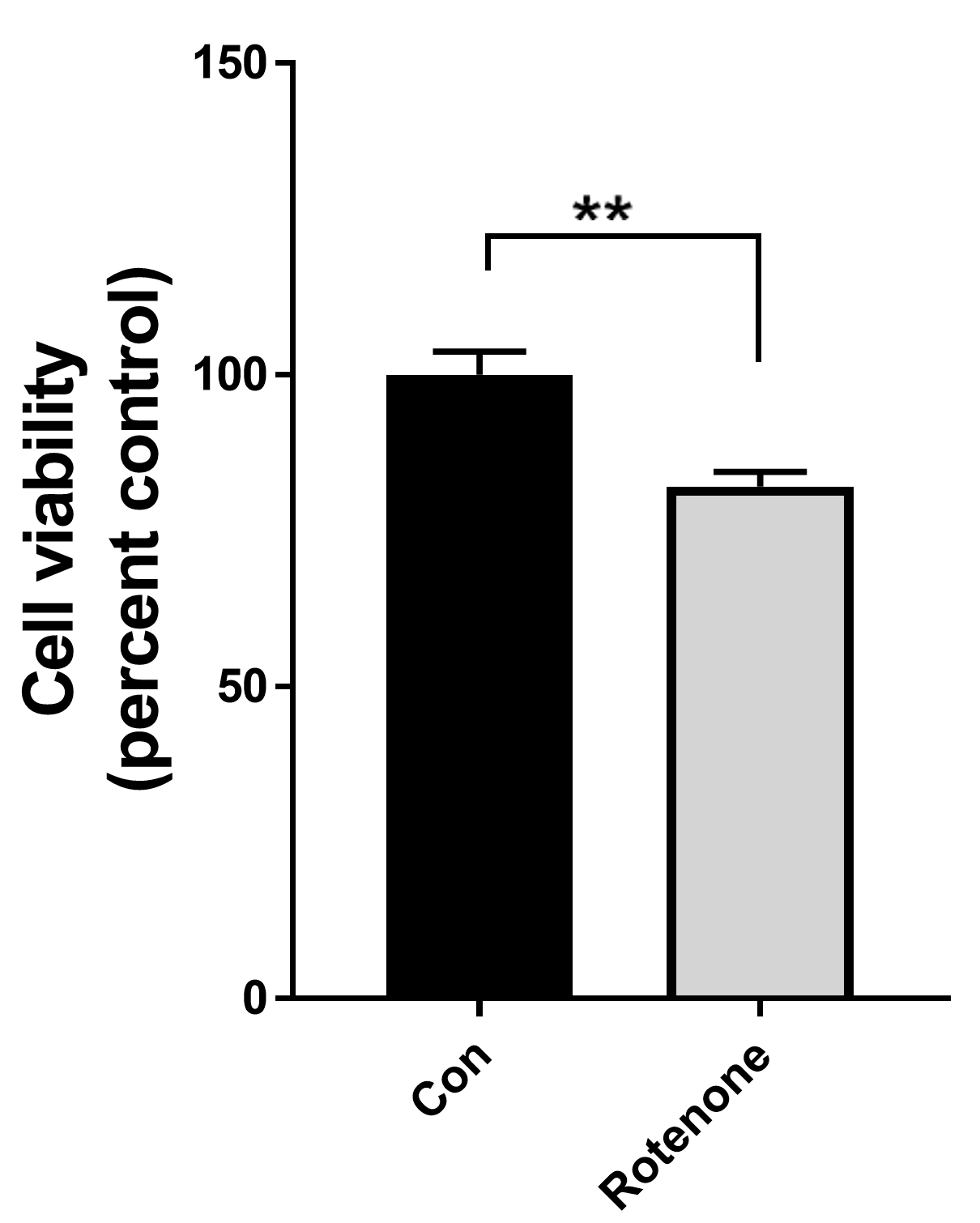

Supplement: Supplementary file 1 [file antioxidants-12-00582-s001.zip › Figure S2.tif]
